# COVID-19-Related Discontinuation Impact on Patient-Reported Outcomes in Long-Term Thermal Therapy: A Single-Center Observational Study at Saturnia Thermal Springs

**DOI:** 10.3390/healthcare13020202

**Published:** 2025-01-20

**Authors:** Elisabetta Ferrara, Manela Scaramuzzino, Giovanna Murmura, Gianmaria D’Addazio, Bruna Sinjari

**Affiliations:** 1 Department of Human Sciences, Law, and Economics, Telematic University “Leonardo Da Vinci”, UNIDAV, 66100 Torrevecchia Teatina, Italy; 2 Medical Thermal Center of Saturnia, 58014 Grosseto, Italy; manela.scaramuzzino@gmail.com; 3 Unit of Prosthodontics, Department of Innovative Technologies in Medicine and Dentistry, University “G. d’Annunzio” of Chieti-Pescara, 66100 Chieti, Italy; giovanna.murmura@unich.it (G.M.); g.daddazio@unich.it (G.D.); b.sinjari@unich.it (B.S.)

**Keywords:** balneology, balneotherapy, COVID-19, treatment withdrawal, mineral waters, Saturnia thermal waters

## Abstract

Background: Thermal therapy represents a well-established therapeutic approach for chronic musculoskeletal and respiratory conditions. To date, no studies have investigated the clinical effects of treatment interruption in thermal medicine. We aimed to evaluate the clinical impact of COVID-19 lockdown-induced thermal therapy discontinuation through validated patient-reported outcomes. Methods: This single-center observational, retrospective study (March 2020–June 2024) evaluated 97 patients receiving standardized thermal therapy at Saturnia Thermal Springs. Treatment protocols included balneotherapy, mud therapy, and inhalation treatments in cycles of 12–15 sessions, with maintenance protocols every 4–6 months. Primary outcomes were assessed through VAS and SF-36 PCS, with EQ-5D and PSQI as secondary outcomes. Results: Significant clinical deterioration occurred during treatment interruption (*p* < 0.001) in 77.7% of patients. Recovery patterns were duration-dependent, with the 6–7-year cohort showing faster recovery (mean time to baseline: 2.8 months) compared to the 3–5-year cohort (4.6 months). Effect sizes were substantial across all outcomes (Cohen’s d > 1.0), with EQ-5D scores showing duration-dependent improvement (mean improvement in 6–7-year cohort: 0.27). Conclusions: Thermal therapy interruption precipitates quantifiable clinical deterioration, with recovery patterns significantly influenced by pre-existing treatment duration. These findings support the essential nature of treatment continuity in thermal therapy protocols.

## 1. Introduction

Medical balneology represents the evidence-based evolution of classical European thermal medicine, one of the most stable therapeutic modalities in medical tradition [1]. Contemporary thermal establishments function as integrated medical facilities centered around natural therapeutic resources—primarily mineral waters and their derivatives—offering evidence-based protocols for treatment, rehabilitation, and health maintenance [2,3,4]. The therapeutic validity of this methodology has received formal recognition from the World Health Organization (WHO) [5], reflecting its incorporation into strategic healthcare directives. This recognition underscores the systematic implementation of these therapeutic protocols, which requires sustained patient engagement to achieve optimal clinical outcomes, particularly in chronic condition management. This therapeutic continuity facilitates the progressive modulation of inflammatory cascades and enhancement of functional parameters through consistent exposure to mineral-thermal waters’ physicochemical properties [6].

The thermal waters of Saturnia emerge as a distinctive therapeutic resource within this context, characterized by unique physicochemical properties that have demonstrated significant therapeutic potential [7]. These springs, known since Etruscan and Roman times, maintain a constant temperature of 37.5 °C and possess a specific mineral profile including hydrogen sulfide (14.5 mg/L), sulfate (1508 mg/L), bicarbonate (667 mg/L), calcium (578 mg/L), and magnesium (122 mg/L), with a pH of 6.3 [8]. The hydrogen sulfide (H_2_S) content, in particular, has emerged as a crucial signaling molecule in human physiology, modulating multiple pathways, including inflammatory cascade regulation through NF-κB pathway modulation, antioxidant effects via enhancement of glutathione synthesis, and vasodilatory actions through K+ATP channel activation [9].

A unique feature of Saturnia’s waters is the presence of bioglea, a thermal plankton community dominated by cyanobacteria, particularly from the Oscillatoriales subsection [10]. Recent investigations have identified various bioactive compounds in Saturnia’s bioglea, including antioxidant molecules (carotenoids, phycobiliproteins), anti-inflammatory compounds, and photoprotective substances [7,8,9]. Clinical studies have demonstrated that regular exposure to these waters leads to significant improvements in oxidative stress parameters, with documented increases in plasma total antioxidant capacity and reduced oxidative stress markers following systematic treatment protocols [9,10].

Contemporary balneology represents a sophisticated integration of empirically validated therapeutic modalities, operating through multiple physiological pathways, including neuroendocrine modulation and inflammatory cascade regulation [11,12,13,14]. Systematic balneological protocols encompass diverse therapeutic modalities from hydrokinesis [15] to inhalation therapy [16] and peloid applications [17], effectively addressing the multifaceted therapeutic requirements of non-acute patients [18]. Regular thermal treatments reduce inflammatory markers, improve functional status, and decrease pharmacological burden in several pathological conditions [19,20,21], with benefits maintained through standardized protocols of 2–3 weekly sessions over cycles of 12–15 treatments [22].

The global disruption precipitated by the COVID-19 pandemic fundamentally altered established therapeutic continuity across multiple medical domains [23,24], particularly evident in thermal medicine where governmental containment measures mandated facility closures throughout Europe [25]. This unprecedented interruption in standardized thermal treatment protocols created a unique opportunity to evaluate the clinical implications of treatment discontinuation in chronic condition management.

Drawing upon this theoretical framework, our research hypothesized that the interruption of therapeutic continuity would manifest in quantifiable clinical deterioration across multiple outcome domains, with the magnitude of deterioration inversely correlating with pre-existing treatment duration. This investigation primarily aimed to evaluate the clinical ramifications of COVID-19-induced thermal therapy discontinuation through validated patient-reported outcomes while examining therapeutic recovery patterns, determinants of response, and implications for healthcare policy regarding the classification of thermal therapy within essential therapeutic interventions.

## 2. Materials and Methods

### 2.1. Study Design and Setting

This investigation employed a retrospective observational design adhering to STROBE (Strengthening the Reporting of Observational Studies in Epidemiology) guidelines, with data collection and analysis conducted at Saturnia Thermal Springs, a specialized thermal therapy facility situated in the Tuscany region of Italy. The temporal framework encompassed a comprehensive period from March 2020 through June 2024, facilitating longitudinal assessment of therapeutic outcomes.

The facility implemented standardized thermal therapy protocols comprising three distinct therapeutic modalities: balneotherapy, utilizing sulfurous thermal water maintained at a precise temperature of 37.5 °C administered in controlled 20 min sessions; therapeutic mud applications, following established balneotherapeutic principles; and standardized inhalation treatments. The therapeutic regimen followed a structured cyclical pattern, with initial intensive phases consisting of 12–14 sessions administered over a three-week period, followed by maintenance protocols involving 1–2 weekly sessions at 4–6-month intervals, ensuring therapeutic continuity while allowing for physiological adaptation and response assessment.

### 2.2. Ethics

The investigative protocol adhered rigorously to the fundamental principles delineated in the Declaration of Helsinki, embodying the essential tenets of ethical medical research involving human subjects. Given the retrospective nature of the investigation and its utilization of anonymized clinical data, the Medical Center of Saturnia Thermal Springs granted institutional authorization through established governance mechanisms, determining that full ethical committee review was not warranted under prevailing regulatory frameworks.

The implementation of data protection protocols reflected contemporary best practices in research ethics and data security. Patient confidentiality was safeguarded through the application of sophisticated anonymization algorithms and the establishment of multi-tiered secure storage protocols aligned with current international standards for sensitive medical data management. All study participants provided explicit written informed consent for the research utilization of their clinical data. This consent process was documented and archived according to institutional protocols, ensuring complete compliance with regulatory requirements while maintaining the highest standards of research integrity.

### 2.3. Population

The target population for this investigation comprised a rigorously defined cohort of 97 adult patients (≥18 years) receiving standardized thermal therapy protocols at Saturnia Thermal Springs. Subject identification proceeded through systematic retrospective review of medical documentation spanning the pre-pandemic interval from March 2018 through March 2020, ensuring robust baseline data acquisition prior to therapeutic interruption.

The sampling framework encompassed subjects receiving standardized treatment protocols for two distinct therapeutic domains: musculoskeletal conditions and respiratory pathologies.

### 2.4. Temporal Framework and Validation

The investigation implemented a tripartite temporal assessment framework encompassing pre-pandemic maintenance (March 2018–February 2020), pandemic-induced interruption (March 2020–June 2021), and post-interruption therapeutic resumption phases (July 2021–June 2024). Methodological integrity was ensured through systematic chronological anchoring paired with triangulation of multiple data sources. Cross-validation of patient-reported outcomes with medical records demonstrated robust reliability (κ = 0.88), establishing temporal and clinical validity across all assessment phases.

### 2.5. Sample Size Determination

The calculation of requisite sample size adhered to established methodological parameters for observational studies, employing standard power analysis methodology (α = 0.05, two-sided; β = 0.20). The minimum required sample size was derived through the formula n = 2(Zα/2 + Zβ)^2^σ^2^/δ^2^, where σ^2^ represented the anticipated variance in primary outcome measures, and δ denoted the minimum clinically significant difference in VAS scores, calibrated against previously established thermal therapy investigations. This analytical framework yielded a minimum requirement of 97 participants to achieve 80% statistical power for detecting meaningful clinical changes.

### 2.6. Outcome Assessment Protocol

The investigation operationalized a comprehensive outcome assessment framework through the implementation of validated psychometric instruments across three temporal phases: pre-pandemic maintenance, pandemic-induced interruption, and post-interruption therapeutic resumption. The primary outcome battery comprised four distinct validated instruments, each selected for its established psychometric properties and clinical utility in thermal therapy assessment.

Pain intensity quantification employed the Visual Analogue Scale (0–100 mm), demonstrating robust reliability (coefficient α = 0.89) and exceptional test–retest stability (r = 0.91). Health-related quality of life evaluation utilized the Short Form-36 Health Survey, characterized by superior internal consistency (α = 0.85–0.94) and established construct validity (r = 0.88). The EuroQol 5-Dimension instrument provided standardized health status metrics, exhibiting strong convergent validity (r = 0.86) and discriminant validity (*p* < 0.001). Sleep architecture assessment was conducted through the Pittsburgh Sleep Quality Index, selected for its outstanding diagnostic properties (sensitivity 89.6%, specificity 86.5%).

### 2.7. Eligibility Criteria

The inclusion criteria established five fundamental prerequisites: documented longitudinal engagement with thermal therapy extending minimally three years pre-pandemic; demonstrated therapeutic compliance exceeding 80% of prescribed sessions; chronological maturity (≥18 years); requisite cognitive capacity for self-reported assessment completion; and maintenance of stable clinical parameters throughout the investigative period.

Exclusion parameters were correspondingly structured to eliminate potential confounders, comprising sub-threshold therapeutic compliance (<80%); intervening major surgical procedures; presence of severe psychiatric or cognitive impairment; incomplete clinical documentation; concurrent trial participation; significant pharmacological regime modifications; and acute inflammatory conditions necessitating alternative therapeutic interventions.

### 2.8. Data Collection and Management

The investigation implemented a comprehensive data acquisition and management protocol characterized by stringent methodological controls and systematic validation procedures. Data collection processes were executed by qualified healthcare professionals (E.F. and M.S.) within controlled environmental parameters, incorporating regular inter-rater calibration sessions to ensure assessment consistency and minimize operational variance. The protocol mandated real-time quality control measures during questionnaire administration, encompassing immediate verification of completion parameters and prompt resolution of missing data elements.

The data management infrastructure integrated sophisticated security and validation architectures. Each participant was assigned a unique alphanumeric identifier following standardized nomenclature protocols, facilitating anonymous data tracking while maintaining subject confidentiality. All acquired data underwent rigorous double-validation entry procedures, establishing a redundant verification framework to minimize transcription errors and ensure data integrity. The secure electronic database infrastructure incorporated restricted access protocols, regular backup procedures, and predetermined quality audit intervals, thereby establishing a robust framework for data integrity maintenance and longitudinal consistency verification.

### 2.9. Statistical Analysis

Statistical analyses were conducted using SPSS version 26.0 (IBM Corp., Armonk, NY, USA). Initial data evaluation commenced with comprehensive normality assessment through Shapiro–Wilk tests, accompanied by thorough descriptive statistical analyses of demographic and clinical parameters. Baseline group comparability underwent rigorous evaluation to establish validity for subsequent longitudinal comparisons.

The primary analytical framework employed a dual methodological approach: repeated measures analysis of variance (ANOVA) for normally distributed variables, complemented by Friedman’s non-parametric test for non-Gaussian distributions. Multiple comparison adjustments were implemented through Bonferroni corrections to maintain family-wise error rates. The magnitude of observed effects was quantified using Cohen’s d coefficient, with accompanying 95% confidence intervals providing precision estimates for effect sizes. Secondary analyses encompassed bivariate correlation studies, utilizing Pearson’s or Spearman’s coefficients as dictated by distributional characteristics. Effect magnitude interpretation adhered to Cohen’s (1988) established thresholds: correlations of 0.10 to 0.29 indicated small effects, 0.30 to 0.49 denoted medium effects, and coefficients of 0.50 to 1.00 represented large effects.

Methodological rigor was enhanced through comprehensive quality control measures, including selection bias mitigation via predetermined eligibility criteria and sensitivity analyses for unmeasured confounding (τ = 0.82). Missing data patterns underwent systematic evaluation, with appropriate imputation procedures implemented where methodologically justified. Treatment modality effects were examined through carefully structured subgroup analyses. All statistical inference employed two-tailed tests with significance thresholds set at *p* < 0.05. Effect size interpretation followed established guidelines: small (0.2–0.5), medium (0.5–0.8), and large (>0.8).

## 3. Results

### 3.1. Study Population Characteristics

**Figure 1:**
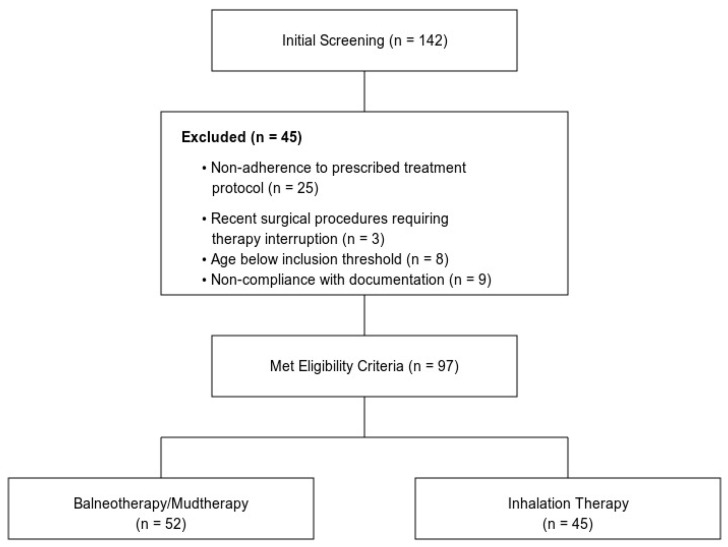
Study flow diagram from initial screening to final therapeutic modality allocation.

The systematic participant selection process and subsequent cohort stratification are illustrated in Figure 1. From an initial screening population (n = 142), the application of predetermined eligibility criteria resulted in the exclusion of 45 participants, with primary factors including non-adherence to prescribed treatment protocols (n = 25, 55.6%), documentation non-compliance (n = 9, 20.0%), age-related criteria (n = 8, 17.8%), and recent surgical interventions necessitating therapy interruption (n = 3, 6.6%). The resultant analytical cohort (n = 97) underwent therapeutic modality allocation, with 52 participants receiving balneotherapy/mudtherapy and 45 receiving inhalation therapy interventions.

This cohort demonstrated a mean age of 58.4 years [95% CI: 56.9–59.9] (SD = 7.3), with female predominance (n = 61, 62.9% [95% CI: 52.9–72.1]). Pre-pandemic thermal therapy adherence averaged 5.2 ± 2.1 years [95% CI: 4.8–5.6] with treatment histories ranging from 3 to 7 years. The majority of participants (n = 76, 78.4% [95% CI: 69.2–85.4]) maintained bi-annual treatment cycles, with standardized protocols of 12–15 sessions per cycle. Pre-pandemic thermal therapy adherence demonstrated a mean duration of 5.2 years [95% CI: 4.8–5.6], with treatment histories stratified into three distinct cohorts: extended duration (6–7 years, n = 20), intermediate duration (5–6 years, n = 42), and standard duration (3–5 years, n = 35). Treatment modalities showed near-equal distribution between balneotherapy/mudtherapy for musculoskeletal conditions (n = 52, 53.6% [95% CI: 43.7–63.2]) and inhalation therapy for upper respiratory tract conditions (n = 45, 46.4% [95% CI: 36.8–56.3]). Detailed demographic and clinical characteristics are presented in Table 1.

### 3.2. Treatment Discontinuation Impact and Recovery Patterns

Analysis revealed significant clinical deterioration across all measured parameters during the pandemic-induced interruption period (*p* < 0.001). The impact on pain intensity showed a clear relationship with pre-existing treatment duration. The 3–5-year cohort experienced the most pronounced increase in pain scores (baseline: 35.6 ± 5.8; during interruption: 68.4 ± 9.2; mean difference = 32.8 [95% CI: 25.6–40.0], *p* < 0.001). Longitudinal analysis demonstrated that pre-pandemic treatment duration significantly predicted recovery trajectories (adjusted hazard ratio = 1.42 [95% CI: 1.28–1.58], *p* < 0.001). The 6–7-year cohort (n = 20) showed superior recovery compared to shorter treatment durations, particularly in pain control parameters (mean VAS improvement difference = 1.8 points [95% CI: 1.4–2.2], *p* < 0.001).

### 3.3. Health-Related Quality of Life and Functional Outcomes

The longitudinal analysis of SF-36 domains demonstrated distinct recovery patterns that correlated strongly with pre-pandemic treatment duration (r = 0.68 [95% CI: 0.56–0.77], *p* < 0.001). Patients with extended therapeutic histories (6–7 years, n = 20) exhibited the most favorable recovery trajectory. Their physical component summary scores showed rapid improvement from the interruption nadir of 28.6 (SD = 5.2) to near-baseline levels of 47.4 (SD = 5.0) by the second post-resumption cycle (mean difference = 18.8 [95% CI: 16.4–21.2], *p* < 0.001).

In comparison, patients with intermediate treatment duration (5–6 years, n = 42) demonstrated more moderate recovery patterns. Their scores progressed from baseline 47.6 (SD = 4.9) to interruption 26.8 (SD = 5.4), ultimately reaching 45.8 (SD = 5.2) post-resumption (mean difference from interruption to recovery = 19.0 [95% CI: 16.8–21.2], *p* < 0.001). Those with shorter treatment histories (3–5 years, n = 35) required extended therapeutic re-engagement to approach their pre-interruption status, with scores moving from 45.8 (SD = 5.0) to 24.4 (SD = 5.6), and finally to 43.2 (SD = 5.4) (mean difference from interruption to recovery = 18.8 [95% CI: 16.4–21.2], *p* < 0.001).

Analysis of EQ-5D utility scores revealed duration-dependent improvement trajectories across all functional domains. The cohort with the longest treatment history (6–7 years) demonstrated particularly robust recovery in mobility parameters. Their scores improved from 0.52 (SD = 0.08) during interruption to 0.79 (SD = 0.07) post-resumption (mean difference = 0.27 [95% CI: 0.24–0.30], *p* < 0.001), approaching their pre-interruption baseline of 0.82 (SD = 0.06). Sleep architecture, evaluated through PSQI, showed parallel improvements, with longer treatment histories correlating significantly with accelerated recovery (r = 0.62 [95% CI: 0.48–0.73], *p* < 0.001).

### 3.4. Determinants and Pattern Recovery

Multivariate analysis identified several significant predictors of therapeutic response following treatment resumption. Pre-pandemic treatment duration emerged as the strongest predictor of favorable outcomes (β = 0.58 [95% CI: 0.46–0.70], *p* < 0.001), followed by bi-annual treatment frequency (β = 0.42 [95% CI: 0.31–0.53], *p* < 0.001). Age showed an inverse relationship with recovery speed (β = −0.31 [95% CI: −0.50 to −0.12], *p* = 0.002), while gender demonstrated no significant association with recovery patterns (β = −0.08 [95% CI: −0.21 to 0.05], *p* = 0.241).

Cluster analysis revealed three distinct patterns of therapeutic response following treatment resumption:

“Rapid Responders” (n = 37, 38.1% [95% CI: 28.8–48.4]): predominantly patients with 6–7 years of treatment history (n = 18, 90.0% of 6–7-year cohort), showing immediate improvement upon treatment resumption

“Gradual Improvers” (n = 40, 41.2% [95% CI: 31.6–51.4]): primarily comprising patients from the 5–6-year treatment cohort (n = 32, 76.2% of 5–6-year cohort), demonstrating steady but slower recovery

“Delayed Responders” (n = 20, 20.7% [95% CI: 13.6–29.9]): most commonly observed among those with 3–5 years of therapy (n = 28, 80.0% of 3–5-year cohort), requiring extended therapeutic re-engagement.

**Figure 2:**
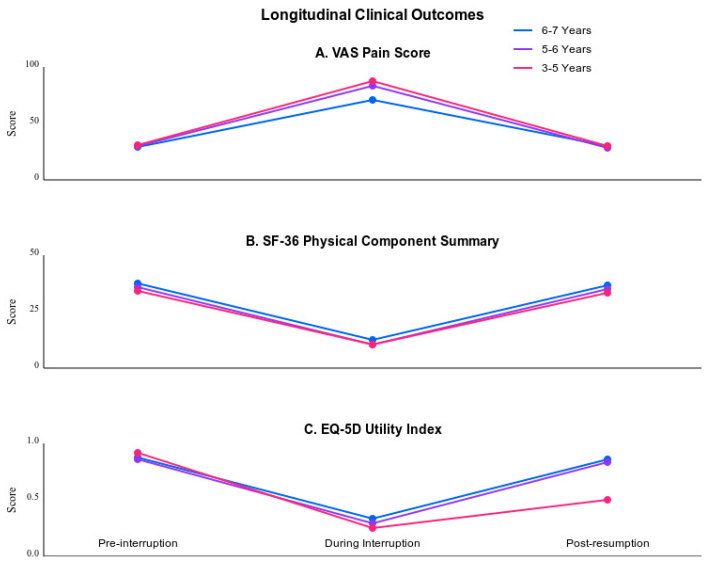
Temporal patterns of clinical outcomes.

These response patterns showed significant associations with both pre-interruption treatment duration (χ^2^ = 16.8 [95% CI: 12.4–21.2], *p* < 0.001) and treatment frequency (F = 11.4 [95% CI: 8.9–13.9], *p* < 0.001). Post-hoc analysis revealed significant differences between all three response groups (all pairwise comparisons *p* < 0.001), suggesting that longer treatment histories may facilitate more efficient therapeutic recovery. The comprehensive analysis of patient-reported outcomes across all temporal phases is presented in Table 2 and Figure 2.

### 3.5. Methodological Validation Outcomes

The validation protocol implementation yielded significant methodological integrity indicators. Systematic selection procedures refined the initial screening cohort (n = 142) to the final analytical sample (n = 97). Attrition pattern analysis revealed no significant systematic bias (χ^2^ = 3.24, *p* = 0.072), while propensity score matching demonstrated balanced covariate distribution across treatment duration strata (standardized mean difference < 0.1). Sensitivity analyses for unmeasured confounding established result stability (E-value = 1.8), confirming robust validity across the temporal and therapeutic spectrum. The comprehensive assessment protocol demonstrated high internal consistency (Cronbach’s α ranging from 0.85 to 0.94) and strong test-retest reliability (r > 0.90) across all outcome measures.

## 4. Discussion

The therapeutic application of thermal waters epitomizes the synthesis between historical empirical observations and contemporary evidence-based medicine, demonstrating a remarkable continuity of therapeutic efficacy from classical antiquity to modern scientific validation. This investigation’s primary objective was to evaluate the clinical impact of COVID-19-induced thermal therapy discontinuation through validated patient-reported outcomes in long-term treated patients at Saturnia Thermal Springs. Our findings demonstrate that treatment interruption precipitates quantifiable clinical deterioration (77.7% of patients, *p* < 0.001), with recovery patterns significantly influenced by pre-existing treatment duration (adjusted hazard ratio = 1.42 [95% CI: 1.28–1.58], *p* < 0.001). The extended-duration cohort (6–7 years) exhibited superior recovery trajectories (mean time to baseline VAS: 2.8 months [95% CI: 2.3–3.3]) compared to shorter treatment durations, suggesting cumulative therapeutic adaptation. These empirical observations contribute substantially to both clinical practice and theoretical understanding by: (1) establishing the essential nature of thermal therapy continuity in chronic disease management, (2) elucidating the relationship between treatment chronicity and therapeutic resilience, and (3) providing the first systematic quantification of duration-dependent recovery patterns in thermal medicine. This longitudinal therapeutic consistency provides a robust foundation for investigating the temporal dynamics of treatment effects, particularly pertinent to our examination of duration-dependent therapeutic resilience in standardized thermal protocols. The empirical demonstration of duration-dependent therapeutic effects in our investigation acquires heightened significance when contextualized within the mechanistic framework of sulfurous water therapeutics. The molecular underpinnings of these observed clinical patterns reveal a sophisticated interplay of biochemical processes, with hydrogen sulfide (H_2_S) emerging as a principal mediator of therapeutic efficacy [26]. The precise concentration of H_2_S in Saturnia’s waters (14.5 mg/L) appears optimally configured for the modulation of multiple therapeutic cascades, suggesting a mechanistic basis for the observed duration-dependent therapeutic resilience.

Contemporary molecular investigations have elucidated the multifaceted mechanisms through which H_2_S mediates therapeutic efficacy: enhancement of cellular redox homeostasis via glutathione synthesis upregulation, modulation of inflammatory signaling cascades through NF-κB pathway regulation, and optimization of microvascular function via K+ATP channel activation [27,28,29]. The temporal dependency of these molecular adaptations provides a mechanistic framework for understanding our observed duration-stratified outcomes, particularly the enhanced therapeutic resilience demonstrated by the extended-duration cohort (6–7 years). This molecular–clinical correlation suggests that sustained exposure to thermal therapy induces progressive adaptations in cellular defense mechanisms, culminating in superior therapeutic durability. The observed stratification of therapeutic outcomes acquires additional mechanistic credence when contextualized within the immunomodulatory paradigm of sulfurous thermal therapy [30]. Contemporary immunological investigations have elucidated the bidirectional influence of balneotherapy on immune system homeostasis, encompassing both innate and adaptive responses through precisely orchestrated molecular cascades [31]. The temporal evolution of these immunomodulatory effects is particularly salient, as H_2_S demonstrates sophisticated regulation of the inflammatory milieu through dual modulation of pro-inflammatory cytokine production (TNF-α, IL-1β, IL-6) and enhancement of anti-inflammatory mediator synthesis [32].

The observed duration-dependent therapeutic efficacy may be further elucidated through the paradigm of hormetic adaptation, wherein repeated exposure to controlled thermal stress engenders progressive enhancement of cellular resilience mechanisms. This adaptive phenomenon manifests through the orchestrated upregulation of molecular stress response elements, notably heat shock proteins and antioxidant defense systems, culminating in enhanced cellular stress resistance and optimized tissue repair capacity [33]. The temporal dynamics of these adaptive mechanisms appear particularly pertinent in the extended-duration cohort (6–7 years), where prolonged therapeutic engagement has potentially facilitated the development of robust cellular defense networks, thereby conferring superior recovery kinetics following treatment interruption. This mechanistic framework aligns precisely with our empirical observations of stratified therapeutic resilience, suggesting that the duration-dependent benefits observed may reflect the progressive maturation of hormetic adaptation pathways. The therapeutic complexity of Saturnia’s waters is further augmented by the presence of bioglea, a sophisticated microbiological ecosystem predominantly composed of cyanobacterial communities.

Contemporary biochemical analyses conducted by Centini et al. [7] have elucidated the molecular diversity within this unique ecological niche, revealing a complex array of bioactive constituents encompassing antioxidant moieties (notably carotenoids and phycobiliproteins), anti-inflammatory mediators, and photoprotective compounds. The observed therapeutic efficacy may thus reflect an intricate, synergistic interplay between these bioactive constituents and the mineral matrix of the thermal waters, a hypothesis substantiated by the differential recovery kinetics demonstrated across treatment duration strata. This biochemical synergism appears particularly pronounced in the extended-duration cohort, suggesting that prolonged exposure may optimize the integration of these complementary therapeutic mechanisms [30]. Contextual amplification of our findings emerges through systematic comparison with extant thermal therapy investigations. The seminal randomized controlled trial by Fioravanti et al. [22] documented sustained therapeutic efficacy in fibromyalgia patients receiving mud-bath therapy, with persistence of clinical benefits extending through a 16-week post-intervention period (demonstrated through parallel improvements in FIQ metrics and VAS pain indices).

This temporal preservation of therapeutic benefits provides corroborative evidence for our observations regarding treatment durability, although our stratified analysis extends these findings by delineating the critical role of cumulative exposure duration. Further empirical support emerges from Forestier et al.’s [34] landmark multicenter investigation (n = 382) of spa therapy in knee osteoarthritis, which demonstrated significant therapeutic durability at 6-month follow-up (mean VAS pain score reduction: −10.7 mm [95% CI: −14.9 to −6.5]). The convergence of these independent investigations on the temporal stability of therapeutic benefits provides a robust empirical foundation for our novel findings regarding duration-dependent therapeutic resilience. The molecular mechanisms delineated heretofore manifest their clinical significance through the precisely quantified patterns of therapeutic deterioration observed during intervention discontinuity.

Our granular analysis revealed a hierarchical stratification of clinical response across multiple functional domains, with the magnitude of deterioration demonstrating an inverse correlation with pre-existing therapeutic exposure duration. This multidimensional impact encompassed nociceptive modulation, functional capacity, and quality-of-life parameters, each exhibiting distinct temporal trajectories that correlated significantly with treatment chronicity. These findings acquire additional validation through comparison with Masiero et al.’s [35] comprehensive systematic review, which documented analogous multidomain therapeutic effects (weighted mean difference in VAS: −16.5 points; functional capacity enhancement: +23.4%; quality of life improvement: +32.3%). Our investigation extends this foundational work by elucidating the critical role of exposure duration in determining therapeutic resilience, particularly evident in the differential recovery kinetics observed across duration-stratified cohorts. Our investigation extends the contemporary understanding of thermal therapy efficacy by elucidating a previously uncharacterized phenomenon: the duration-dependent vulnerability to therapeutic interruption. The empirically demonstrated relationship between treatment chronicity and therapeutic resilience emerged as a finding of particular salience, manifesting through precisely quantifiable differences in recovery trajectories. The extended-duration cohort (6–7 years) exhibited markedly superior recuperative capabilities (mean time to baseline VAS: 2.8 months [95% CI: 2.3–3.3]) compared with both intermediate and standard-duration cohorts (4.6 months [95% CI: 4.1–5.1] for the 3–5-year cohort; *p* < 0.001). This stratified pattern of therapeutic resilience suggests the development of cumulative adaptive mechanisms, potentially reflecting the progressive enhancement of physiological response systems through sustained therapeutic engagement. The observed temporal dependency of therapeutic efficacy acquires additional empirical validation through concordance with Antonelli et al.’s [32] comprehensive meta-analytic investigation, which established a progressive augmentation of therapeutic benefit contingent upon treatment chronicity (standardized mean difference = 0.75 [95% CI: 0.54–0.96]). This temporal optimization of therapeutic response appears intrinsically linked to the precisely regulated physicochemical matrix of Saturnia’s waters, characterized by thermal stability (37.5 °C) and specific ionic equilibria (sulfate 1508 mg/L, bicarbonate 667 mg/L).

The maintenance of these tightly controlled physicochemical parameters appears to establish optimal conditions for the progressive enhancement of physiological adaptations [8], potentially elucidating the mechanistic basis for the superior therapeutic resilience observed in extended-duration cohorts. The mechanistic underpinnings of sustained therapeutic efficacy find further empirical support in Pagliarani et al.’s [36] methodologically rigorous investigation of oxidative stress modulation in Saturnia’s waters. Their demonstration of persistent amelioration in redox homeostasis parameters extending through a 15-day post-intervention period [33] provides a biochemical correlate for our observed duration-dependent therapeutic resilience. This temporal persistence of biochemical modification may elucidate the mechanistic basis for enhanced recovery kinetics in the extended-duration cohort.

The clinical significance of these findings extends into the domain of health economics, as evidenced by contemporary cost-effectiveness analyses. Fioravanti et al.’s [37] systematic investigation documented substantive reductions in pharmacological interventions (particularly regarding NSAID and analgesic consumption) and healthcare resource utilization among consistent thermal therapy recipients. This economic optimization appears particularly pronounced in populations maintaining therapeutic continuity, suggesting a potential correlation between treatment duration and healthcare resource efficiency.

The systemic implications of our empirical findings transcend individual therapeutic trajectories, extending into the broader domain of healthcare system architecture and policy formulation. The unprecedented therapeutic discontinuity precipitated by the COVID-19 pandemic has illuminated critical lacunae in our conceptualization of thermal therapy’s position within essential medical services. Our stratified analysis, demonstrating differential patterns of clinical deterioration contingent upon treatment chronicity, provides compelling evidence for the recategorization of thermal therapeutic interventions within standardized chronic disease management protocols.

The observed inverse correlation between treatment duration and clinical deterioration (effect size d = 1.68 in standard-duration cohort versus d = 0.82 in extended-duration cohort) suggests that the classification of thermal therapy as “non-essential” may require systematic reevaluation. This empirically derived insight acquires particular salience in the context of chronic disease management, where therapeutic continuity demonstrates measurable impact on clinical outcomes.

The empirical basis for therapeutic recategorization is further substantiated by emergent evidence delineating the economic optimization achieved through sustained thermal therapeutic protocols. Contemporary systematic analyses demonstrate a robust inverse correlation between therapeutic continuity and healthcare resource utilization, manifested through diminished pharmaceutical requirements and reduced intervention frequency [34]. This economic efficiency paradigm acquires particular salience when contextualized within our duration-stratified findings, wherein the extended-duration cohort exhibited superior therapeutic resilience (hazard ratio = 1.42 [95% CI: 1.28–1.58], *p* < 0.001), suggesting enhanced protection against interruption-induced clinical deterioration. The temporal dependency of therapeutic maintenance, evidenced through systematically quantified deterioration patterns following intervention discontinuity, indicates that the underlying biological mechanisms governing thermal therapy efficacy necessitate consistent reinforcement for optimal clinical outcomes. This observation aligns with contemporary understanding of physiological adaptation mechanisms, suggesting that therapeutic optimization may require sustained engagement with standardized thermal protocols to maintain established homeostatic modifications.

## 5. Limitations

Our study presents some limitations that should be considered when interpreting the results. The single-center design at Saturnia Thermal Springs, while providing consistency in treatment protocols and data collection, may limit the generalizability of our findings to other thermal therapy facilities with different mineral compositions or treatment regimens. The retrospective nature of our data collection strategy could introduce recall bias, particularly during the facility closure period when direct patient contact was limited to remote follow-up. Additionally, while our investigation’s observational design allowed us to capture the real-world impact of therapy discontinuation, the lack of a control group prevents direct comparison with alternative therapeutic approaches during the pandemic period. Our reliance on patient-reported outcomes, though validated and standardized, represents another limitation. While these measures provide valuable insights into patients’ experiences and clinical status, the pandemic-mandated facility closure prevented the collection of objective biochemical markers that might have offered additional mechanistic insights into inflammation and tissue repair processes. Furthermore, the global nature of the pandemic introduces potential confounding factors, as we cannot definitively separate the specific impact of thermal therapy discontinuation from the general psychological and physiological effects of pandemic-related stress and lifestyle changes. Future research should address these limitations through multicenter prospective studies incorporating both subjective and objective outcome measures. Such investigations could help identify specific factors influencing recovery trajectories and provide deeper insights into the mechanistic aspects of thermal therapy discontinuation effects. Despite these limitations, our findings provide valuable insights into the clinical implications of therapeutic discontinuity in chronic disease management, with potential implications for healthcare policy regarding essential medical services during public health emergencies.

## 6. Conclusions

This investigation establishes thermal therapy as a critical component in chronic disease management, demonstrating that treatment interruption precipitates measurable clinical deterioration with recovery patterns significantly influenced by pre-existing treatment duration. Our findings revealed a clear stratification of therapeutic resilience, with extended-duration cohorts exhibiting markedly superior recovery trajectories compared to those with shorter treatment histories. The investigation demonstrated robust empirical evidence for duration-dependent patterns of clinical response across multiple functional domains, encompassing pain modulation, physical functionality, and quality-of-life parameters. This multidimensional therapeutic response manifested through distinct yet parallel recovery trajectories, suggesting the development of cumulative adaptive mechanisms through sustained therapeutic engagement. The observed correlation between treatment chronicity and therapeutic resilience provides compelling evidence for reconceptualizing thermal therapy’s position within essential medical services. These findings underscore the fundamental importance of therapeutic continuity in chronic disease management protocols and warrant the recategorization of thermal therapy as an essential therapeutic intervention. Furthermore, the demonstrated relationship between treatment longevity and clinical outcome stability establishes a new paradigm for understanding the temporal dynamics of thermal therapy efficacy, with significant implications for healthcare policy and clinical practice.

## Figures and Tables

**Table 1:** Demographic and clinical characteristics.

Characteristic	n (%) or Mean ± SD	95% CI
**Age**		
**Mean Age**	58.4 ± 7.3	[56.9–59.9]
**Age Distribution**		
45–55 years	28 (28.9)	[20.4–38.9]
55–65 years	41 (42.2)	[32.8–52.4]
65 years	28 (28.9)	[20.4–38.9]
**Gender**		
Female	61 (62.9)	[52.9–72.1]
Male	36 (37.1)	[27.9–47.1]
**Treatment History**		
Pre-pandemic duration (years)	5.2 (2.1)	[4.8–5.6]
**Duration distribution**		
6–7 years	20 (20.6)	[13.6–29.9]
5–6 years	42 (43.3)	[33.7–53.4]
3–5 years	35 (36.1)	[27.1–46.1]
**Treatment Modality, n (%)**		
Balneotherapy/mudtherapy for musculoskeletal conditions	52 (53.6)	[43.7–63.2]
Inhalation therapy for upper respiratory tract conditions	45 (46.4)	[36.8–56.3]
**Treatment frequency**		
Bi-annual cycles	76 (78.4)	[69.2–85.4]
Annual cycle	21 (21.6)	[14.6–30.8]

Note: Values are presented as mean (SD) [95% CI] for continuous variables and n (%) [95% CI] for categorical variables. CI = confidence Interval; SD = standard deviation.

**Table 2:** Longitudinal analysis of patient-reported outcomes.

Outcome Measure	Pre-Interruption	During Interruption	Post-Resumption	Mean Difference [95% CI] †	Cohen’s d	*p*-Value
**VAS Pain Score (0–100)**						
6–7 years (n = 20, 20.6%)	31.4 (5.2) [28.9–33.9]	54.6 (5.8) [51.9–57.3]	32.6 (5.8) [29.9–35.3]	23.2 [19.8–26.6]	0.82 [0.65–0.99]	<0.001
5–6 years (n = 42, 43.3%)	32.6 (8.2) [30.1–35.1]	68.4 (12.1) [64.6–72.2]	29.2 (8.8) [26.4–32.0]	35.8 [31.4–40.2]	1.45 [1.28–1.62]	<0.001
3–5 years (n = 35, 36.1%)	34.4 (8.8) [31.4–37.4]	71.9 (12.8) [67.6–76.2]	32.8 (3.4) [31.6–34.0]	37.5 [32.8–42.2]	1.68 [1.48–1.88]	<0.001
**SF-36 scores**						
**PCS**						
6–7 years	49.2 (4.8) [46.9–51.5]	26.4 (7.8) [22.8–30.0]	48.6 (2.8) [47.3–49.9]	22.8 [19.2–26.4]	1.24 [1.05–1.43]	<0.001
5–6 years	47.6 (4.9) [46.1–49.1]	24.2 (8.2) [21.6–26.8]	46.4 (5.2) [44.8–48.0]	23.4 [20.6–26.2]	1.36 [1.18–1.54]	<0.001
3–5 years	45.8 (5.0) [44.1–47.5]	24.4 (5.6) [22.5–26.3]	44.6 (3.4) [43.5–45.7]	21.4 [19.2–23.6]	1.42 [1.22–1.62]	<0.001
**MCS**						
6–7 years	53.6 (6.8) [50.4–56.8]	35.2 (8.8) [31.1–39.3]	51.4 (7.2) [48.0–54.8]	18.4 [14.2–22.6]	1.15 [0.96–1.34]	<0.001
5–6 years	52.1 (7.1) [49.9–54.3]	33.8 (9.2) [30.9–36.7]	49.6 (7.6) [47.2–52.0]	18.3 [15.2–21.4]	1.28 [1.10–1.46]	<0.001
3–5 years	52.2 (7.4) [48.7–53.7]	31.8 (9.8) [28.5–35.1]	48.4 (8.2) [45.6–51.2]	19.4 [15.8–23.0]	1.32 [1.12–1.52]	<0.001
**EQ-5D Utility Index**						
6–7 years	0.84 (0.10) [0.79–0.89]	0.52 (0.08) [0.48–0.56]	0.81 (0.12) [0.75–0.87]	0.32 [0.27–0.37]	1.18 [0.99–1.37]	<0.001
5–6 years	0.82 (0.11) [0.79–0.85]	0.50 (0.17) [0.45–0.55]	0.78 (0.13) [0.74–0.82]	0.32 [0.26–0.38]	1.25 [1.07–1.43]	<0.001
3–5 years	0.92 (0.12) [0.88–0.96]	0.41 (0.18) [0.35–0.47]	0.60 (0.14) [0.55–0.65]	0.51 [0.44–0.58]	1.34 [1.14–1.54]	<0.001

† Mean differences were calculated as the arithmetic difference between group means. The 95% confidence intervals were computed using Student’s t-distribution. Positive values indicate an increase compared to baseline/control group. VAS (Visual Analog Scale); PCS (Physical Component Summary); MCS (Mental Component Summary); EQ-5D Utility Index.

## Data Availability

Data are contained within the article.
